# Succinate Dehydrogenase Subunit D as a Redox-Responsive Mitochondrial Component Linked to Aquaporin-Associated Hydrogen Peroxide Signaling in Glioblastoma Cells

**DOI:** 10.3390/antiox15091125

**Published:** 2026-09-05

**Authors:** Kuen-Jang Tsai, Kuan-Tso Chen, Chin-Chuan Tsai, Zi-Xuan Hong, Li-Ying Qiu, Chan-Chuan Liu, Kwang-Yu Chang, Pin-Yuan Chen, Chia-Hung Chien

**Affiliations:** 1 Department of Surgery, E-Da Cancer Hospital, Kaohsiung 82445, Taiwan; tsai560612@gmail.com; 2 Department of Surgery, E-Da Dachang Hospital, Kaohsiung 80706, Taiwan; 3 College of Medicine, I-Shou University, Kaohsiung 82445, Taiwan; ed108780@edah.org.tw (K.-T.C.); tsaicc@isu.edu.tw (C.-C.T.); 107721101@gms.tcu.edu.tw (Z.-X.H.); 4 Department of Chinese Medicine, E-Da Cancer Hospital, Kaohsiung 82445, Taiwan; 5 The School of Chinese Medicine for Post-Baccalaureate, I-Shou University, Kaohsiung 82445, Taiwan; 6 Department of Chinese Medicine, E-Da Dachang Hospital, Kaohsiung 80706, Taiwan; 7 School of Medicine, I-Shou University, Kaohsiung 82445, Taiwan; 8 National Institute of Cancer Research, National Health Research Institutes, Tainan 70456, Taiwan; f0561012@gmail.com (L.-Y.Q.); sln4421@hotmail.com (C.-C.L.); kwang2@nhri.edu.tw (K.-Y.C.); 9 Department of Pharmacology, College of Medicine, National Cheng Kung University, Tainan 70101, Taiwan; 10 Department of Oncology, National Cheng Kung University Hospital, College of Medicine, National Cheng Kung University, Tainan 70101, Taiwan; 11 Department of Neurosurgery, Chang Gung Memorial Hospital at Keelung, Keelung 204201, Taiwan; pinyuanc@cgmh.org.tw; 12 School of Medicine, Chang Gung University, Taoyuan 333323, Taiwan; 13 Department of Neurosurgery, Chang Gung Memorial Hospital at Linkou, Taoyuan 333423, Taiwan

**Keywords:** glioblastoma, SDHD, hydrogen peroxide, ROS, aquaporin

## Abstract

Glioblastoma (GBM) frequently recurs after temozolomide (TMZ) therapy and exhibits substantial redox plasticity. Our previous work showed that the effects of hydrogen peroxide (H_2_O_2_) vary with its level and between parental and TMZ-resistant GBM cells. Succinate dehydrogenase subunit D (SDHD), a membrane-anchoring component of mitochondrial complex II, is positioned at the interface of electron transport and redox homeostasis, but its regulation in GBM remains unclear. We therefore examined whether SDHD expression changes across distinct H_2_O_2_-responsive contexts involving aquaporins (AQPs) and AKT. TCGA transcriptomic analysis showed higher SDHD mRNA expression in WHO grade IV than in grade II/III gliomas, whereas paired primary/recurrent high-grade glioma samples showed heterogeneous SDHD changes at recurrence. TMZ reduced SDHD, and SDHD knockdown decreased intracellular reactive oxygen species. Combined redox perturbation reduced SDHD, whereas AKT inhibition restored SDHD expression. Under receptor-associated signaling conditions, EGFRvIII expression or CXCL12 stimulation increased measured H_2_O_2_ together with AQP3, AKT Ser473 phosphorylation, and SDHD. In TMZ-resistant cells, pharmacological perturbation of aquaporin-associated signaling decreased AQP9 together with AKT Ser473 phosphorylation and SDHD. An AQP3-targeting compound further reduced cell density when combined with TMZ. Together, these findings suggest that changes in SDHD expression are consistent with a compensatory redox response in GBM cells and that its regulation varies with the nature of H_2_O_2_-associated signaling during TMZ-related stress.

## 1. Introduction

Glioblastoma (GBM) is the most common aggressive primary malignant brain tumor in adults. Despite maximal safe resection followed by radiotherapy with concomitant and adjuvant temozolomide (TMZ), recurrence is common and long-term survival remains poor [1]. Acquired resistance is accompanied by substantial metabolic and redox adaptation. In our previous studies, TMZ-resistant GBM models showed enrichment of superoxide dismutase 2 (SOD2) and SH3GLB1-associated mitochondrial remodeling [2,3]. More recently, we demonstrated that hydrogen peroxide (H_2_O_2_) acts as a level- and cell-state-dependent signal and signaling-range increases can support AKT-associated adaptive responses, whereas stronger oxidative burden can produce an opposing response, particularly in TMZ-resistant cells [4]. These findings prompted us to examine whether a mitochondrial respiratory-chain component is also regulated across distinct redox states.

Reactive oxygen species (ROS) are not merely toxic by-products of metabolism; at controlled levels, they also act as signaling mediators that regulate proliferation, migration, stemness, and stress adaptation [5]. GBM cells therefore face a redox balance in which signaling-range ROS can support oncogenic and adaptive pathways, whereas excessive ROS may trigger apoptosis, ferroptosis, or other forms of cell death [6]. TMZ has been reported to increase ROS production and activate downstream stress responses [7], while resistant glioma cells can reinforce antioxidant defenses and remodel mitochondrial metabolism to keep ROS within a tolerable range [8,9]. Thus, the biological consequence of H_2_O_2_ depends not only on its presence but also on its magnitude, duration, cellular state, and subcellular source [4].

Succinate dehydrogenase (SDH; mitochondrial complex II) links the tricarboxylic acid cycle to the electron transport chain [10]. The complex contains the catalytic SDHA and SDHB subunits and the membrane-anchoring SDHC and SDHD subunits. SDHD is embedded in the inner mitochondrial membrane and anchors complex II, placing it at a potential interface between electron transport and membrane-associated redox regulation. Alterations in SDH are well established in cancer biology [11,12], and complex II can influence mitochondrial ROS production in a manner that depends on metabolic context [13,14]. We therefore focused on SDHD as a membrane-anchoring component of complex II and examined whether its expression changes under redox-related conditions in GBM cells. In some tumor models, reduced expression or inhibition of SDH components has been associated with altered redox status and stress-adaptive phenotypes [15,16]. These reports provide additional biological rationale for examining SDHD in GBM cells.

A second clue came from peroxiporins, aquaporins that facilitate H_2_O_2_ movement across membranes [17]. AQP3 is located predominantly at the plasma membrane and can facilitate the entry of extracellularly generated H_2_O_2_ during growth-factor- and chemokine-associated signaling, including EGFR and CXCL12/CXCR4 pathways [18,19,20]. Because EGFRvIII is a constitutively active EGFR mutant frequently found in GBM [1], we used EGFRvIII as a GBM-relevant model of sustained EGFR signaling, while CXCL12 was examined as another receptor-associated stimulus. Our group has also reported the biological relevance of CXCL12 signaling in GBM [21]. AQP9 likewise transports H_2_O_2_ in mammalian cells [22], and a short AQP9 isoform has been reported in the inner mitochondrial membrane [23], providing a rationale for examining the relationship between AQP9 and mitochondria-associated redox signaling in TMZ-resistant cells.

Based on these observations, we investigated SDHD expression in glioma and during TMZ-associated stress and examined its relationship with intracellular ROS, aquaporin-associated H_2_O_2_ signaling, and AKT in GBM cells.

## 2. Materials and Methods

### 2.1. Clinical Transcriptomic and Survival Analysis

To assess the clinical association of SDHD expression, transcriptomic data from 620 glioma cases in the TCGA dataset were accessed and analyzed through GlioVis (https://gliovis.bioinfo.cnio.es/) and were analyzed according to the historical WHO grade categories used in the source dataset. Expression values were grouped as grade II, grade III, or grade IV and displayed as log2-transformed values. Because this dataset is based on older WHO grade II–IV classifications, we used it only to compare SDHD expression among the three grade groups and not to infer progression to current WHO-defined IDH-wildtype GBM [24]. Survival curves were obtained from Chinese Glioma Genome Atlas (CGGA) and The Cancer Genome Atlas (TCGA) datasets through GlioVis.

### 2.2. Paired Treatment-Naive and Recurrent High-Grade Glioma Samples and Heatmap Analysis

To compare treatment-naive and recurrent tumors, we examined 14 paired high-grade glioma RNA-sequencing samples for SDH family genes and SDH-associated factors, including SDHA, SDHAF1, SDHAF2, SDHAF3, SDHAF4, SDHB, SDHC, and SDHD. The use of these RNA-sequencing data was approved by the Institutional Review Board of Chang Gung Memorial Hospital (No. 201800077B0) [3], and the dataset has been deposited in the NCBI Gene Expression Omnibus under accession number GSE339484. The original report of this cohort documented molecular and histological heterogeneity, including grade III and IDH1-mutant cases [3]; accordingly, the cohort is referred to here as paired high-grade glioma rather than a molecularly homogeneous set of current-WHO-defined GBM. For each gene, expression in the recurrent sample was divided by the corresponding value in the matched treatment-naive sample and log2-transformed for heatmap visualization. All recurrent tumors were obtained after standard treatment, although the number and course of treatments varied among patients. Accordingly, recurrence-associated expression changes were considered in the setting of prior multiple treatments.

### 2.3. Cell Cultures

Human U87MG and A172 GBM cell lines were used as parental models because matched TMZ-resistant derivatives (U87MGR and A172R) had been generated and characterized in our previous studies, allowing direct within-line comparisons of treatment-sensitive and acquired-resistance states [2,3,4]. TMZ-resistant derivatives were generated by long-term exposure of parental cells to TMZ (100 μM). After an initial reduction in cell proliferation and survival, surviving cells gradually recovered, and resistant clones were subsequently isolated by single-cell cloning, as described previously [2]. U87MG was obtained from the Bioresource Collection and Research Center (Hsinchu, Taiwan), and A172 was obtained from the American Type Culture Collection (Manassas, VA, USA). Cells were maintained in DMEM (GeneDireX, Taoyuan, Taiwan) supplemented with 10% fetal bovine serum (HyClone, Logan, UT, USA) and 1% penicillin/streptomycin (Gibco, Grand Island, NY, USA). TMZ-resistant derivatives were generated by prolonged TMZ selection and maintained under the same basal culture conditions.

### 2.4. Reagents and Treatment Conditions

Cells were exposed to TMZ (Sigma-Aldrich, St. Louis, MO, USA) alone or in combination with redox- or signaling-modulating reagents. The principal reagents included 3-amino-1,2,4-triazole (ATZ; Sigma-Aldrich), 4-hydroxy-2-nonenal (HNE; Cayman Chemical, Ann Arbor, MI, USA), MK-2206 (Cayman Chemical), recombinant human CXCL12 (PeproTech, Cranbury, NJ, USA), HgCl_2_ (Sigma-Aldrich), MG132 (Sigma-Aldrich), sodium diethyldithiocarbamate trihydrate (DETC; Sigma-Aldrich), and the AQP3-targeting inhibitor DFP00173 (MedChemExpress, Monmouth Junction, NJ, USA). TMZ was used at 100 μM, ATZ at 10 mM, HNE at 5 μM, HgCl_2_ at 10 μM, DETC at 500 nM, DFP00173 at 10 μM, and MK-2206 at 5 μM where indicated. ATZ and HNE were used as redox modulators that interfere with H_2_O_2_-detoxifying systems, following the experimental approach established in our previous study [4]. For the TMZ time-course experiment, parental cells were treated for 0, 24, and 48 h before SDHD immunoblotting.

### 2.5. EGFRvIII Overexpression and Gene Knockdown

To examine receptor-associated redox signaling, parental GBM cells expressing empty vector (EV; pHβAPr-1-neo) or EGFRvIII (pHβAPr-1-neo-EGFRvIII) were analyzed for intracellular H_2_O_2_ and downstream protein expression. EGFRvIII and CXCL12 were evaluated as separate upstream stimuli. To examine the contribution of SDHD to intracellular ROS, SDHD-targeting shRNA and scramble shRNA control cells (RNAi Core of Academia Sinica, Taipei, Taiwan) were generated in TMZ-resistant GBM cells and used for DHE fluorescence and immunoblot validation.

### 2.6. Western Blotting

Whole-cell lysates were prepared after the indicated treatments. Equal amounts of protein were separated by sodium dodecyl sulfate–polyacrylamide gel electrophoresis and transferred to membranes for immunoblot analysis. Primary antibodies included anti-SDHD (Abcam, Cambridge, UK), anti-AQP3 (GeneTex, Irvine, CA, USA), anti-AQP9 (Proteintech, Rosemont, IL, USA), anti-phospho-AKT (Ser473; Cell Signaling Technology, Danvers, MA, USA), anti-total AKT (Cell Signaling Technology), anti-phospho-EGFR (Cell Signaling Technology), anti-EGFRvIII (Sigma-Aldrich), anti-OXPHOS cocktail (Abcam), anti-vinculin (Thermo Fisher Scientific, Waltham, MA, USA), and anti-β-actin (Merck Millipore, Burlington, MA, USA). Signals were visualized by chemiluminescence. Where densitometric quantification was performed, target-protein signals were normalized to the indicated loading control before comparison with the reference condition.

### 2.7. DHE Fluorescence Assay for Intracellular ROS

Intracellular ROS levels were assessed using dihydroethidium (DHE; Cayman Chemical, Ann Arbor, MI, USA). A172R scramble and A172R shSDHD cells were labeled with DHE and incubated at 37 °C for 30 min. After washing, cells were resuspended in PBS and analyzed by flow cytometry using a FACSCalibur flow cytometer (BD Biosciences, San Jose, CA, USA). Up to 10,000 cells were collected per sample, and data were analyzed using BD CellQuest Pro software, version 6.0 (BD Biosciences, San Jose, CA, USA). Relative DHE fluorescence was expressed as fold change relative to the scramble control.

### 2.8. Measurement of Intracellular H_2_O_2_

Intracellular H_2_O_2_ concentrations were measured using a commercial H_2_O_2_ detection kit (Cayman Chemical) according to the manufacturer’s instructions. H_2_O_2_ was quantified after EGFRvIII expression or CXCL12 stimulation and normalized to protein content. Results were expressed as μM/μg protein.

### 2.9. Quantitative Real-Time PCR

To evaluate CXCL12 expression after redox modulation, U87MGR and A172R cells were treated with vehicle, TMZ, DETC, or TMZ plus DETC for 24 h. Total RNA was extracted and reverse-transcribed to cDNA before quantitative real-time PCR (qRT-PCR). qRT-PCR was performed using SYBR Green chemistry on an ABI 7000 Sequence Detection System (Applied Biosystems, Foster City, CA, USA). The human CXCL12 primer sequences were ATTCTCAACACTCCAAACTGTGC (forward) and ACTTTAGCTTCGGGTCAATGC (reverse). Relative CXCL12 expression was normalized to GAPDH and calculated using the 2^−ΔΔCt^ method, with the vehicle-treated control used as the calibrator.

### 2.10. Cell Density Assay

GBM cells were plated in 24-well plates at a density of 5 × 10^4^ cells/well and cultured overnight to allow attachment. Cells were then exposed to vehicle, TMZ (100 μM), DFP00173 (10 μM), or TMZ plus DFP00173 for 72 h. Following treatment, cells were stained with 0.5% methylene blue (Sigma-Aldrich) in 50% ethanol for 90 min. Excess dye was removed, incorporated stain was solubilized in 1% N-lauroylsarcosine, and absorbance was measured at 570 nm. Relative cell density was normalized to the vehicle-treated control.

### 2.11. Statistical Analysis

Statistical analyses were performed using Prism 7 (GraphPad, La Jolla, CA, USA). Comparisons between two groups were evaluated using unpaired two-tailed Student’s *t*-tests. Comparisons involving three or more groups were analyzed using one-way analysis of variance (ANOVA), followed by Tukey’s multiple-comparison test. Unless otherwise stated, *n* = 3 denotes three independent biological experiments. A two-sided *p* value < 0.05, 0.01 and 0.001 was considered statistically significant.

## 3. Results

### 3.1. SDHD Expression in TCGA Glioma Data and Paired Primary/Recurrent High-Grade Gliomas

**Figure 1:**
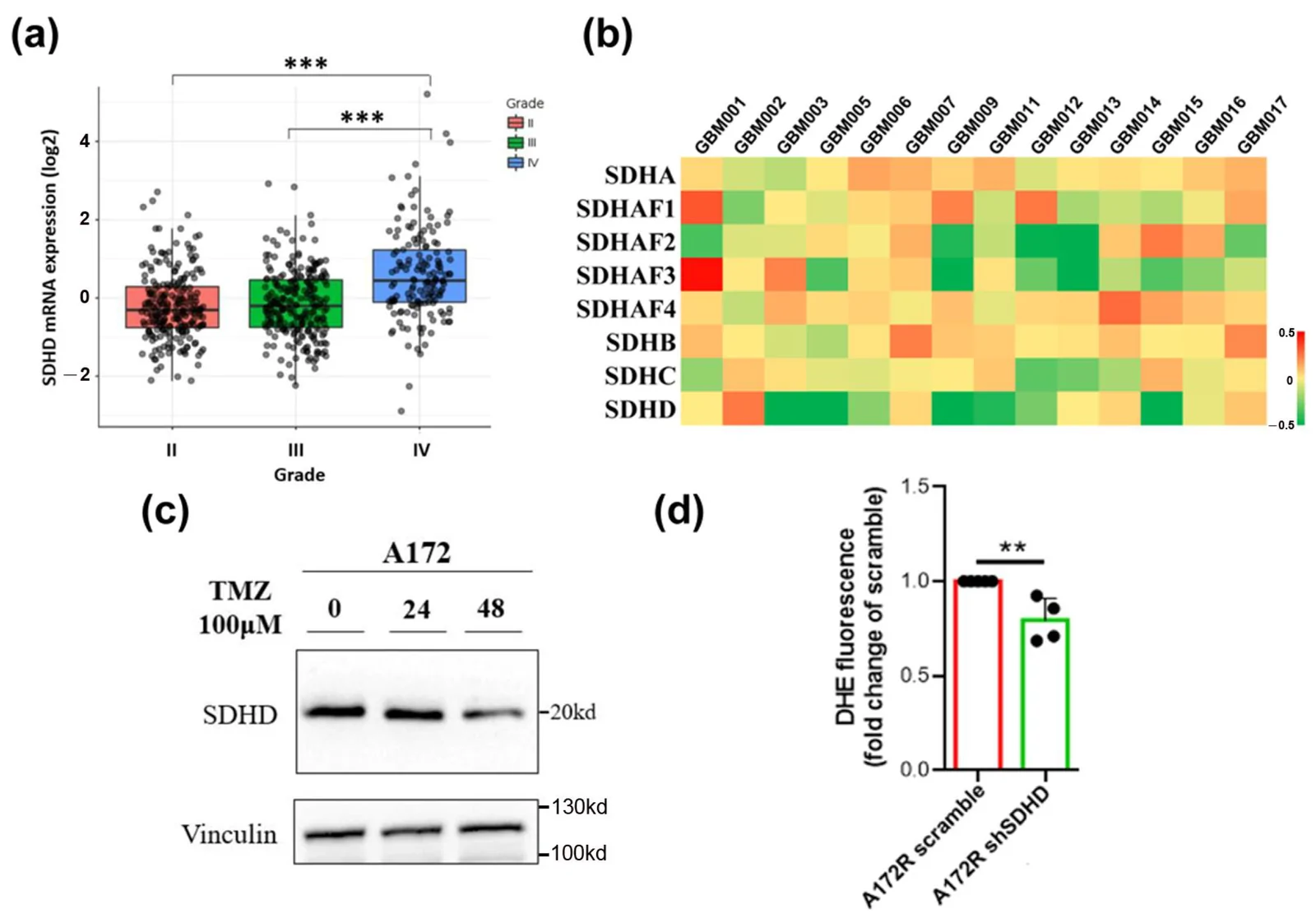
SDHD expression in TCGA gliomas, paired primary/recurrent high-grade gliomas, and intracellular ROS after SDHD depletion. (**a**) SDHD mRNA expression (log2) in WHO grade II (*n* = 226), grade III (*n* = 244), and grade IV (*n* = 150) gliomas from the TCGA dataset accessed through GlioVis. Group comparisons were performed using one-way ANOVA followed by Tukey’s multiple-comparison test; *** *p* < 0.001. (**b**) Heatmap showing relative mRNA expression of SDH-family genes (SDHA, SDHAF1-4, SDHB, SDHC, and SDHD) in 14 paired primary and recurrent high-grade glioma samples. Values represent the log2(recurrent/primary) expression ratio for each gene. (**c**) SDHD protein expression in A172 cells treated with TMZ (100 μM) for 0, 24, and 48 h. (**d**) DHE fluorescence in A172R cells expressing scramble shRNA or shSDHD. Data are presented as fold change relative to scramble control. Two-group comparison was performed using an unpaired two-tailed Student’s *t*-test; ** *p* < 0.01. Cell-based experiments were performed in three independent biological replicates.

To examine the clinical expression pattern of SDHD, we analyzed TCGA glioma transcriptomic data accessed through GlioVis (*n* = 620). SDHD mRNA expression was significantly higher in WHO grade IV tumors than in grade II or grade III tumors (Figure 1a). We next analyzed RNA-seq data from 14 paired primary and recurrent high-grade glioma samples. SDHD expression was lower in 8 recurrent tumors, whereas the remaining paired samples showed stable or increased expression (Figure 1b).

### 3.2. TMZ Decreases SDHD Expression, Whereas SDHD Depletion Reduces Intracellular ROS

To determine whether SDHD expression changes during TMZ exposure, parental A172 cells were treated with TMZ for 0, 24, or 48 h. SDHD protein progressively decreased over time (Figure 1c). We next examined intracellular ROS after SDHD depletion in TMZ-resistant A172R cells. DHE fluorescence was significantly lower in shSDHD cells than in scramble controls (Figure 1d).

### 3.3. Combined TMZ/ATZ/HNE Treatment Reduces SDHD, Whereas MK-2206 Restores SDHD Expression

**Figure 2:**
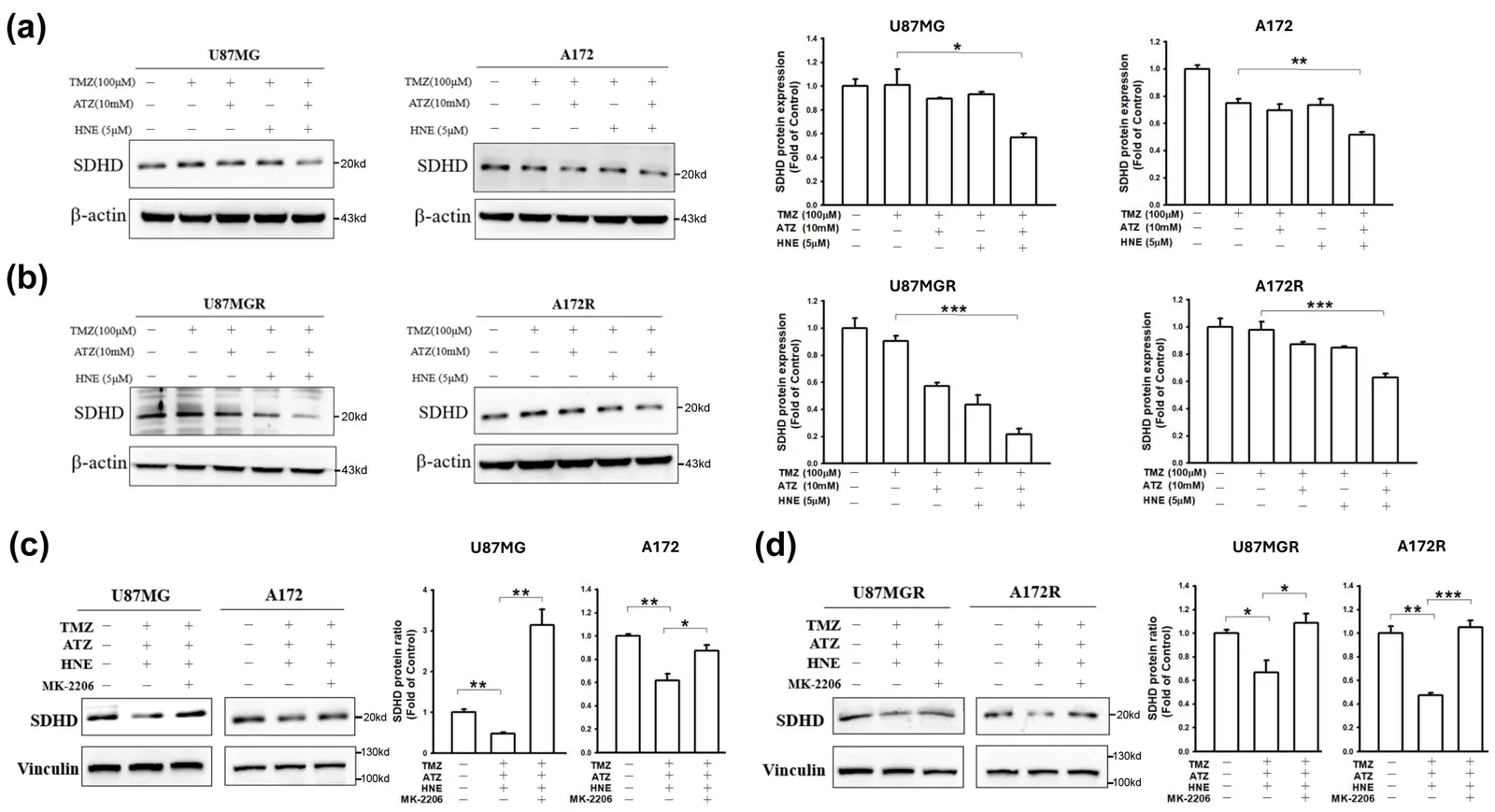
Effects of TMZ/ATZ/HNE treatment and AKT inhibition on SDHD expression in GBM cells. (**a**) Immunoblot analysis of SDHD in parental U87MG and A172 cells treated with TMZ (100 μM), ATZ (10 mM), HNE (5 μM), or the indicated combinations. (**b**) Immunoblots of SDHD in U87MGR and A172R cells under the same treatment conditions. (**c**) Immunoblot analysis of SDHD in parental cells treated with TMZ, ATZ, HNE, and MK-2206 (5 μM) as indicated. (**d**) Immunoblots of SDHD in TMZ-resistant cells under the same treatment conditions. Quantification is shown. Comparisons among treatment groups were performed using one-way ANOVA followed by Tukey’s multiple-comparison test; *** *p* < 0.001, ** *p* < 0.01, * *p* < 0.05. *n* = 3 independent biological experiments.

To examine SDHD expression under the indicated redox-modulating treatments, parental and TMZ-resistant GBM cells were exposed to TMZ, ATZ, HNE, or their combinations. The combined TMZ/ATZ/HNE treatment reduced SDHD expression in parental U87MG and A172 cells and in TMZ-resistant U87MGR and A172R cells (Figure 2a,b). Addition of MK-2206 increased SDHD expression relative to the TMZ/ATZ/HNE condition in both parental and TMZ-resistant cells (Figure 2c,d).

### 3.4. HgCl_2_-Containing Treatment Alters AQP9, AKT Phosphorylation, and SDHD in TMZ-Resistant Cells

**Figure 3:**
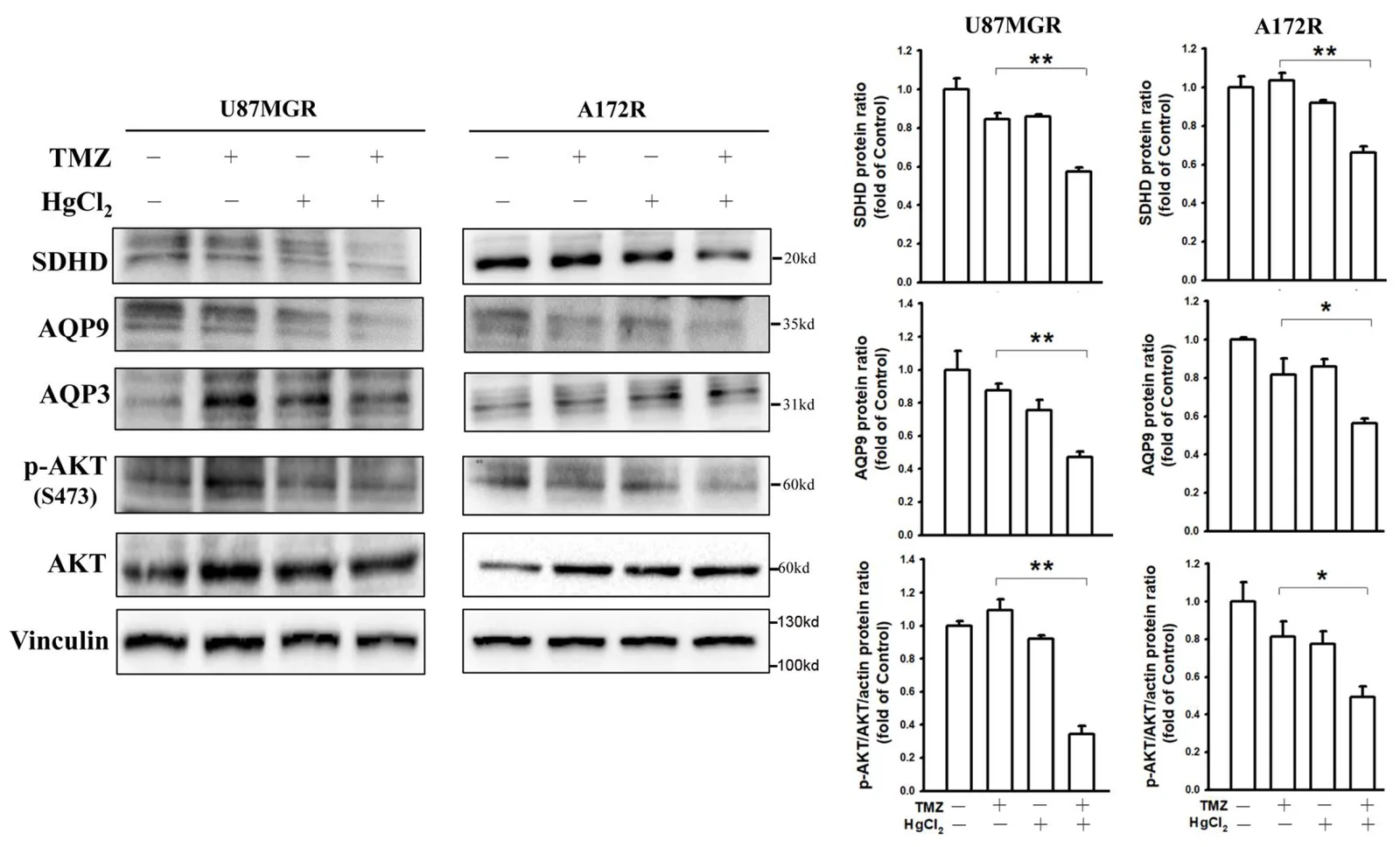
Effects of HgCl_2_-containing treatment on AQP9, AQP3, AKT phosphorylation, and SDHD in TMZ-resistant GBM cells. Immunoblots of SDHD, AQP9, AQP3, p-AKT (Ser473), total AKT, and loading control in U87MGR and A172R cells treated with TMZ, HgCl_2_, or their combination. TMZ: 100 μM; HgCl_2_: 10 μM. Comparisons among treatment groups were performed using one-way ANOVA followed by Tukey’s multiple-comparison test; ** *p* < 0.01, * *p* < 0.05. *n* = 3 independent biological experiments.

Analysis of public datasets showed that higher AQP3 and AQP9 expression was associated with poorer survival in GBM (Figure S1a). We next treated TMZ-resistant U87MGR and A172R cells with TMZ, HgCl_2_, or their combination. HgCl_2_-containing conditions, particularly TMZ plus HgCl_2_, reduced AQP9, p-AKT (Ser473), and SDHD, whereas AQP3 was not comparably reduced (Figure 3). Other respiratory-chain proteins, including SDHB, ATP5A, and NDUFB8, did not show uniform reductions under these conditions (Figure S1b).

### 3.5. EGFRvIII Expression and CXCL12 Stimulation Increase H_2_O_2_, AQP3, p-AKT, and SDHD in Parental Cells

**Figure 4:**
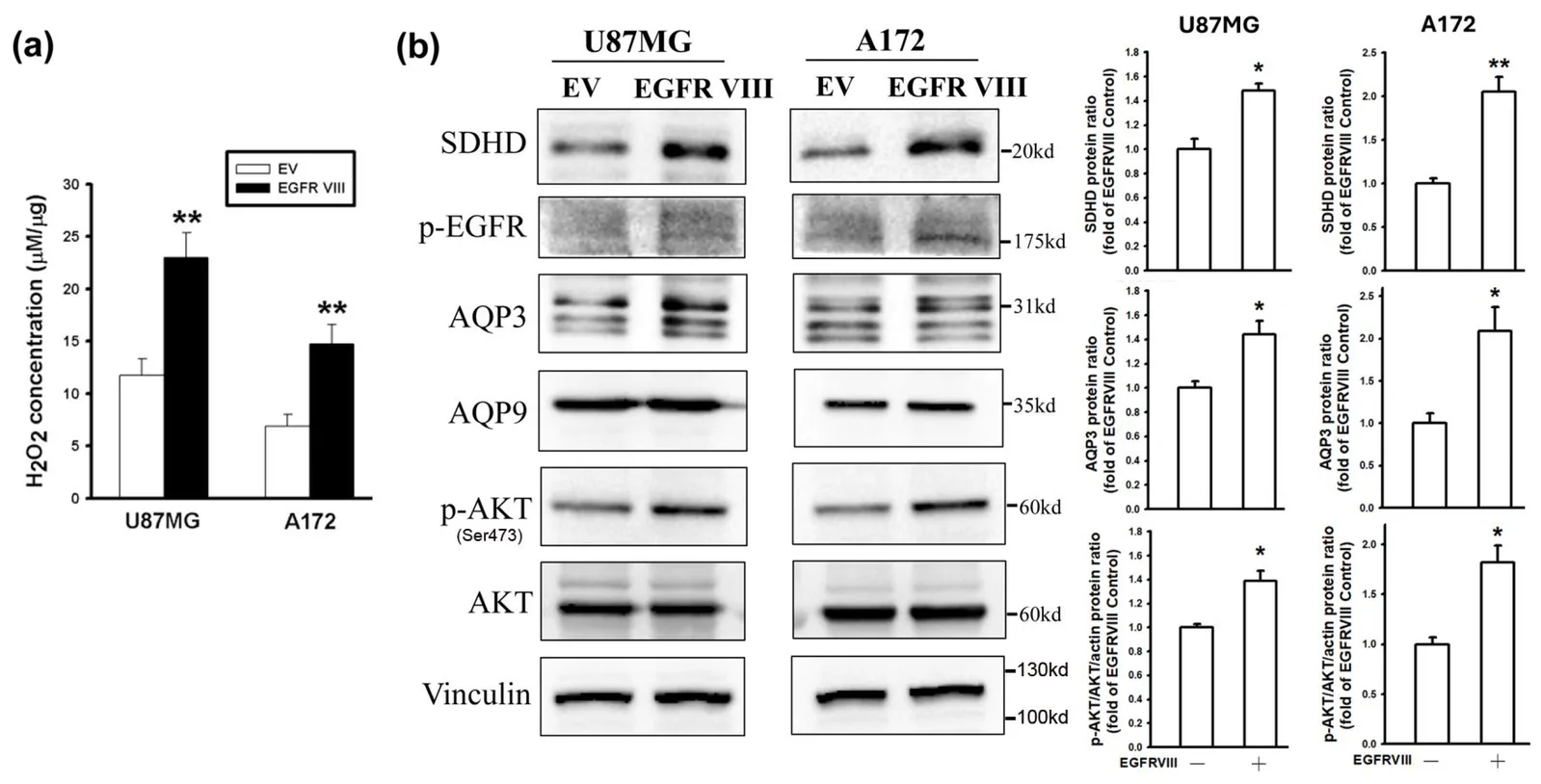
Effects of EGFRvIII expression on intracellular H_2_O_2_, AQP3, p-AKT, and SDHD in parental GBM cells. (**a**) Intracellular H_2_O_2_ concentrations in parental cells expressing empty vector (EV) or EGFRvIII. (**b**) Immunoblots of SDHD, p-EGFR, AQP3, AQP9, p-AKT (Ser473), and total AKT in EV- and EGFRvIII-expressing cells. Two-group comparisons were performed using an unpaired two-tailed Student’s *t*-test; ** *p* < 0.01, * *p* < 0.05. *n* = 3 independent biological experiments.

**Figure 5:**
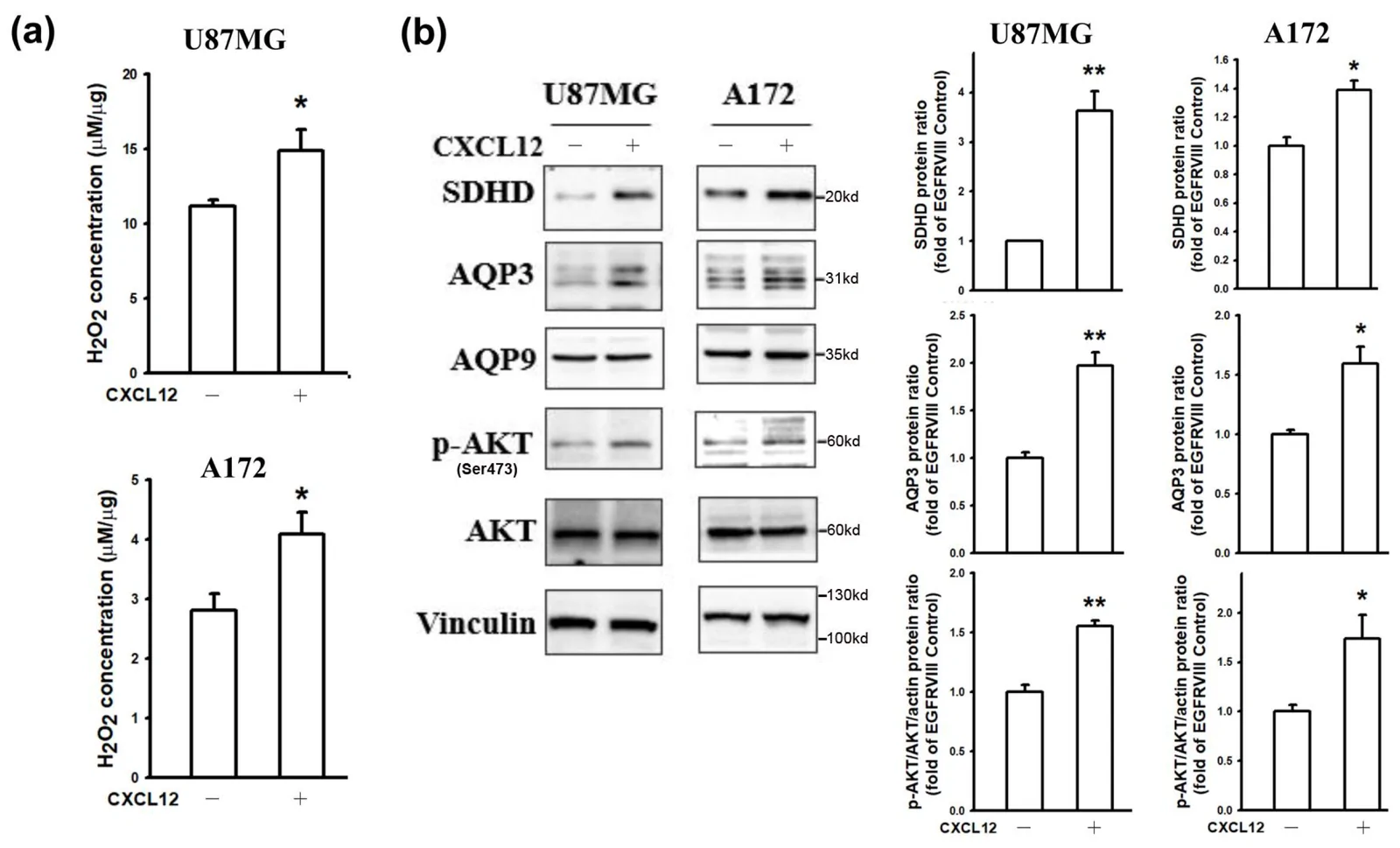
Effects of CXCL12 stimulation on intracellular H_2_O_2_, AQP3, p-AKT, and SDHD in parental GBM cells. (**a**) Intracellular H_2_O_2_ concentrations in parental cells cultured with or without CXCL12. (**b**) Immunoblots of SDHD, AQP3, AQP9, p-AKT (Ser473), and total AKT in parental cells treated with or without CXCL12. CXCL12: 100 ng/mL. Two-group comparisons were performed using an unpaired two-tailed Student’s *t*-test; ** *p* < 0.01, * *p* < 0.05. *n* = 3 independent biological experiments.

To examine receptor-associated H_2_O_2_ signaling, we assessed EGFRvIII expression and CXCL12 stimulation in parental GBM cells. EGFRvIII expression increased SOD2 (Figure S2a) and intracellular H_2_O_2_ (Figure 4a), together with increased p-EGFR, AQP3, p-AKT (Ser473), and SDHD (Figure 4b). CXCL12 stimulation similarly increased intracellular H_2_O_2_ (Figure 5a), AQP3, p-AKT (Ser473), and SDHD (Figure 5b). In TMZ-resistant cells, TMZ increased CXCL12 mRNA, whereas SOD inhibitor (DETC) reduced this increase (Figure S2b).

### 3.6. Proteasome Inhibition Alters SDHD Abundance and Banding Patterns

**Figure 6:**
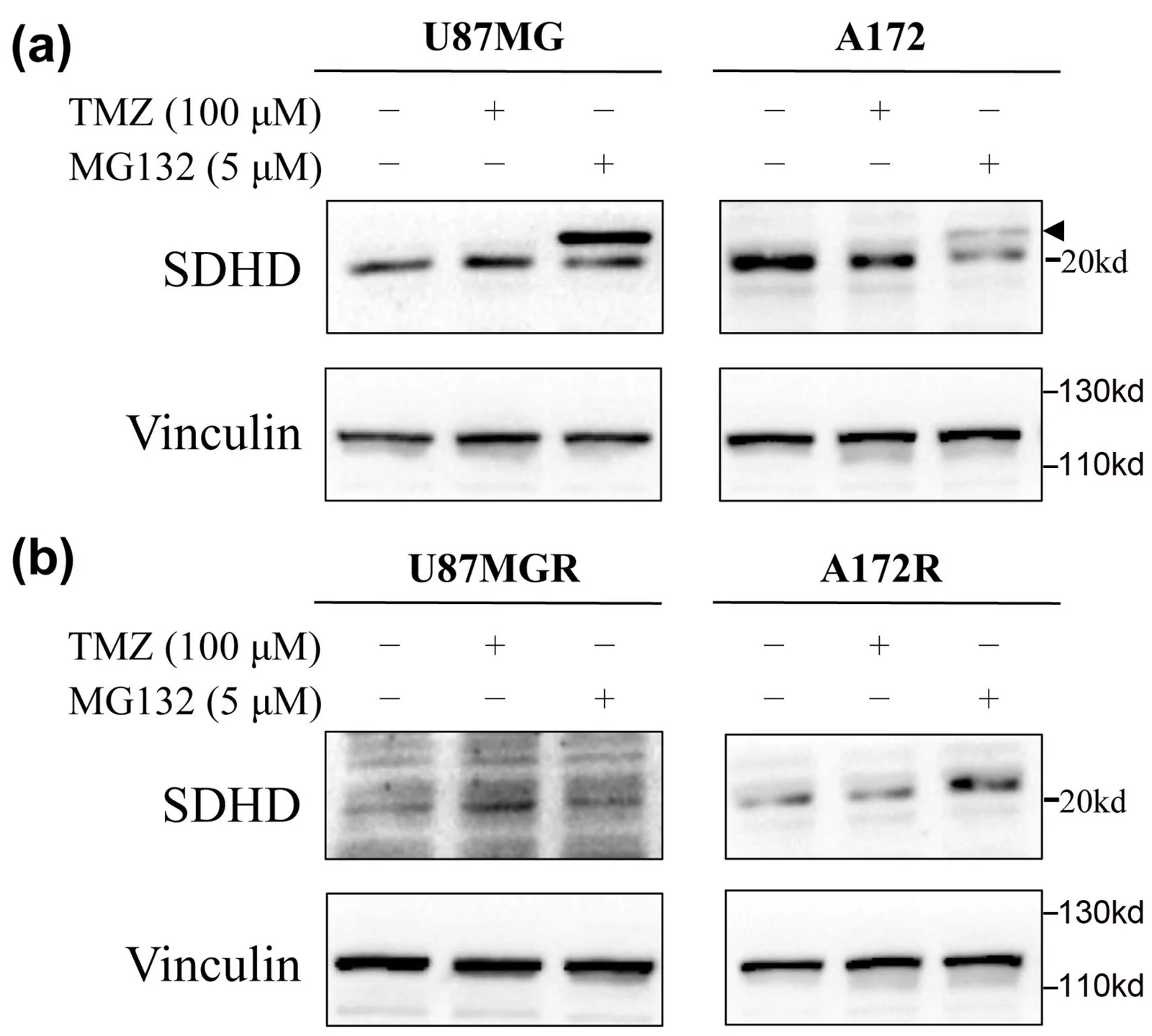
Effects of proteasome inhibition on SDHD abundance and banding patterns. (**a**) Immunoblots of SDHD in parental cells treated with vehicle, TMZ, or MG132. A higher-molecular-weight SDHD-reactive band and the canonical approximately 20kd SDHD band are shown. (**b**) Immunoblots of SDHD in TMZ-resistant cells under the same treatment conditions. TMZ: 100 μM; MG132: 5 μM. *n* = 3 independent biological experiments. The black triangle indicates the higher-molecular-weight SDHD-reactive band.

To examine the effect of proteasome inhibition on SDHD, parental and TMZ-resistant GBM cells were treated with MG132. In parental cells, MG132 produced a prominent higher-molecular-weight SDHD-reactive band above the canonical approximately 20kd species, accompanied by reduced abundance of the canonical band (Figure 6a). In TMZ-resistant cells, MG132 increased the canonical approximately 20kd SDHD band (Figure 6b).

### 3.7. TMZ Combined with DFP00173 Further Reduces GBM Cell Density

**Figure 7:**
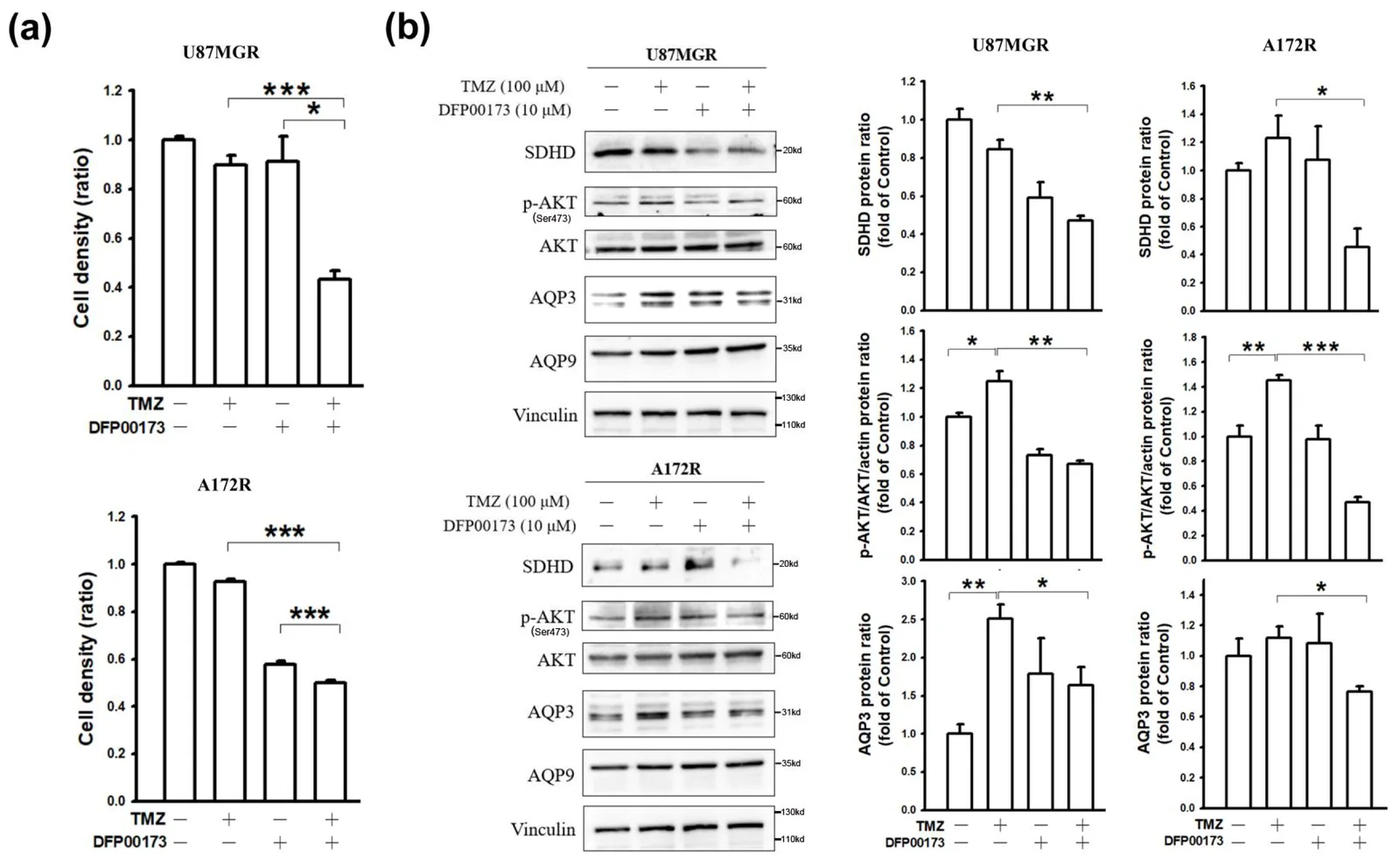
Combined TMZ and DFP00173 treatment reduces GBM cell density. (**a**) Relative cell density after treatment with vehicle, TMZ, DFP00173, or TMZ plus DFP00173 for 72 h. Values are expressed relative to vehicle control. (**b**) Immunoblots of SDHD, p-AKT (Ser473), total AKT, AQP3, and AQP9 in TMZ-resistant cells under the same treatment conditions. TMZ: 100 μM; DFP00173: 10 μM. Comparisons among treatment groups were performed using one-way ANOVA followed by Tukey’s multiple-comparison test; *** *p* < 0.001, ** *p* < 0.01, * *p* < 0.05. *n* = 3 independent biological experiments.

Finally, we examined the effect of DFP00173 on the response to TMZ. In TMZ-resistant cells, combined TMZ and DFP00173 treatment produced a greater reduction in cell density than either treatment alone (Figure 7a). Under the same treatment conditions, the combination was accompanied by lower SDHD, p-AKT (Ser473), and AQP3 levels, whereas AQP9 showed comparatively little change (Figure 7b).

## 4. Discussion

In this study, we examined SDHD as a redox-responsive mitochondrial component associated with H_2_O_2_/ROS and aquaporin-related signaling in GBM cells. SDHD mRNA was higher in TCGA grade IV gliomas than in grade II/III tumors, whereas paired primary/recurrent high-grade gliomas showed heterogeneous SDHD changes. In cell models, TMZ progressively reduced SDHD, and SDHD knockdown lowered intracellular ROS. Combined TMZ/ATZ/HNE treatment reduced SDHD, whereas MK-2206 restored SDHD under this treatment condition. By contrast, EGFRvIII expression and CXCL12 stimulation increased H_2_O_2_ together with AQP3, p-AKT, and SDHD. HgCl_2_-containing conditions in resistant cells produced coordinated decreases in AQP9, p-AKT, and SDHD, and DFP00173 combined with TMZ further reduced cell density. Together, these observations identify SDHD as a redox-responsive component linked to AQP3/AQP9-associated H_2_O_2_ signaling in GBM cells.

SDH connects the TCA cycle with the electron transport chain and can contribute to mitochondrial ROS generation [10,12,14,25]. Mitochondrial metabolic remodeling is also increasingly recognized as a feature of GBM adaptation and therapeutic resistance [26,27]. The higher SDHD expression observed in grade IV TCGA tumors is therefore compatible with increased mitochondrial metabolic and redox demands in aggressive glioma, although SDHD abundance alone does not indicate complex II enzymatic activity. In the paired cohort, SDHD changes at recurrence were heterogeneous rather than uniform. This variability is consistent with the biological diversity of recurrent tumors, but the decrease observed in a subset of paired samples provided a rationale to test SDHD regulation directly under controlled TMZ exposure.

The TMZ time-course and SDHD-knockdown experiments provide complementary evidence linking SDHD to cellular redox status. TMZ progressively decreased SDHD protein, whereas SDHD knockdown reduced DHE fluorescence, showing that experimental reduction in SDHD is accompanied by lower intracellular ROS in this setting. Complex II can generate ROS under both forward and reverse electron flow [14,25], and suppression of SDH components has been reported to reduce oxidative stress and support tumor-cell survival in some settings [15,16]. Our previous study also directly showed increased mitochondrial and cytosolic H_2_O_2_ and ROS following TMZ/ATZ/HNE treatment in these GBM models [4]. However, because ROS/H_2_O_2_ was not measured in parallel with SDHD under all experimental conditions in the present study, the observed decrease in SDHD is best interpreted as being consistent with a compensatory redox response rather than as evidence that SDHD downregulation directly limits ROS accumulation. Antioxidant systems including NRF2 and glutathione may operate in parallel and also contribute to TMZ resistance in GBM [8,9].

The AKT results are best interpreted in relation to the type of redox stimulus. Our previous study showed that H_2_O_2_ signaling in these GBM models varies with oxidative burden and the parental/resistant cell state [4]. In the present study, combined TMZ/ATZ/HNE treatment reduced SDHD and MK-2206 restored SDHD, demonstrating AKT-sensitive regulation under this combined treatment. In contrast, EGFRvIII expression or CXCL12 stimulation increased H_2_O_2_, AQP3, p-AKT, and SDHD in parallel. H_2_O_2_ can function as a second messenger during receptor signaling but can produce different cellular responses when oxidative burden increases [5,17]. Thus, the AKT-SDHD relationship observed during combined oxidative perturbation is not expected to be identical to that seen during receptor-associated H_2_O_2_ signaling.

These findings are also consistent with established roles of peroxiporins in H_2_O_2_ transport. AQP3 facilitates H_2_O_2_ entry during EGFR- and CXCL12-associated signaling [18,19,20], providing a biological basis for the coordinated increases in H_2_O_2_, AQP3, p-AKT, and SDHD observed in our parental GBM cells. AQP9 can also transport H_2_O_2_, and a short AQP9 isoform has been reported in the inner mitochondrial membrane [22,23]. In resistant cells, HgCl_2_ treatment reduced AQP9 together with p-AKT and SDHD. Because HgCl_2_ is thiol-reactive and has been used as a nonselective aquaporin inhibitor [28,29], these coordinated changes support an association between AQP9-related redox signaling and SDHD regulation, although they do not establish that selective AQP9 inhibition directly causes the decrease in SDHD. In addition, HgCl_2_ has been reported to increase mitochondrial H_2_O_2_ generation and promote glutathione oxidation [28], which may also contribute to the reduction in SDHD observed in resistant cells, consistent with the decrease in SDHD under combined redox perturbation in the present study (Figure 2). Finally, the greater reduction in cell density produced by TMZ plus DFP00173 is consistent with the reported AQP3 preference of DFP00173 [30] and with studies showing that AQP3 inhibition or AQP9 depletion can impair tumor-cell fitness in other cancer models [31,32]. These observations support aquaporin-associated H_2_O_2_ handling as a potential modulator of GBM adaptation to TMZ.

The present findings should be interpreted within the scope of the clinical and experimental models used in this study, although several aspects of the clinical data and experimental design limit the extent of mechanistic interpretation. The TCGA grade II–IV comparison is based on older WHO classifications and therefore reflects differences among historically defined glioma groups rather than a biological progression sequence under the current WHO system [24]. Likewise, although the paired recurrent tumors provide clinically relevant evidence of heterogeneous SDHD changes after treatment, differences in individual treatment histories prevent these changes from being attributed specifically to TMZ. The cellular studies used two established GBM models with matched TMZ-resistant derivatives, which enabled direct within-line comparison but cannot capture the full heterogeneity of GBM. The mechanistic scope of the present study is further limited by the absence of specific genetic validation of AQP3 and AQP9, direct functional assessment of Complex II activity and mitochondrial respiration, and in vivo validation of the proposed regulatory relationships. Future studies integrating these approaches will help to further define the contribution of aquaporin-associated H_2_O_2_ signaling to SDHD regulation during GBM adaptation.

## 5. Conclusions

**Figure 8:**
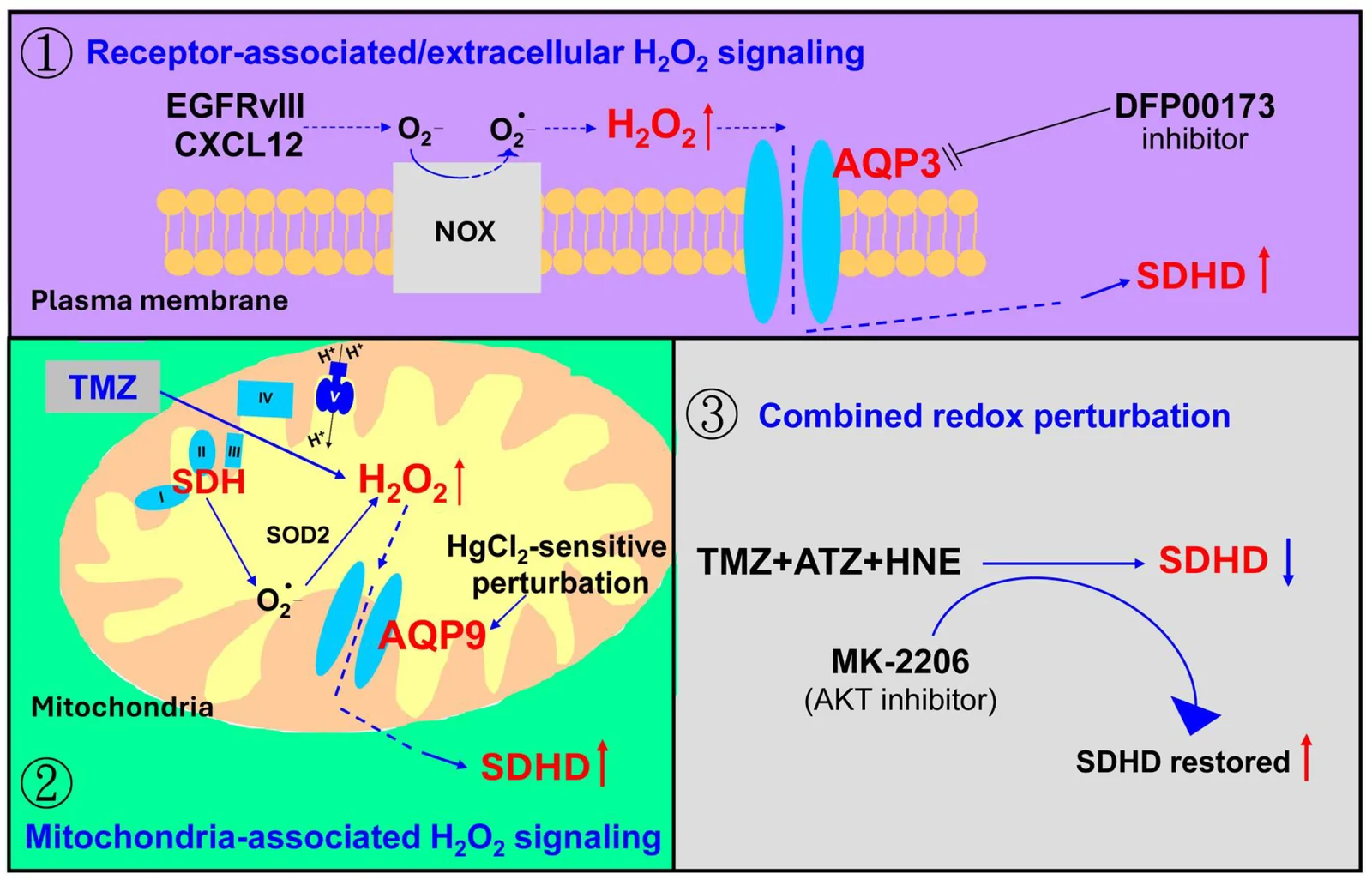
Schematic summary of AQP3/AQP9-associated H_2_O_2_ signaling and SDHD regulation in GBM cells. Receptor-associated signaling induced by EGFRvIII or CXCL12 is associated with increased H_2_O_2_, AQP3, and SDHD expression. Mitochondria-associated AQP9/H_2_O_2_ signaling is linked to SDHD regulation, whereas HgCl_2_ represents a nonselective, HgCl_2_-sensitive perturbation of this signaling condition. Combined treatment with temozolomide (TMZ), 3-amino-1,2,4-triazole (ATZ), and 4-hydroxy-2-nonenal (HNE) decreases SDHD expression, while the AKT inhibitor MK-2206 restores SDHD levels. DFP00173 indicates pharmacological inhibition of AQP3-associated signaling. Solid arrows indicate the indicated relationships, whereas dashed arrows indicate indirect or simplified relationships for which intermediate steps are not shown. T-shaped lines indicate inhibition, and upward/downward arrows indicate increases/decreases.

In conclusion, our findings identify SDHD as a redox-responsive mitochondrial component in GBM cells whose expression changes during TMZ-associated stress and AQP3/AQP9-associated H_2_O_2_ signaling (Figure 8). Together, the results are consistent with SDHD regulation as part of the compensatory redox response of GBM cells and support further investigation of aquaporin-associated H_2_O_2_ signaling during TMZ treatment.

## Data Availability

The original contributions presented in this study are included in the article/Supplementary Material. Further inquiries can be directed to the corresponding author.

## Supplementary Materials

The following supporting information can be downloaded at: https://www.mdpi.com/article/10.3390/antiox15091125/s1.

## Abbreviations

The following abbreviations are used in this manuscript:

AKT	protein kinase B
AQP	aquaporin
AQP3	aquaporin 3
AQP9	aquaporin 9
ATZ	3-amino-1,2,4-triazole
ATP	adenosine triphosphate
CGGA	Chinese Glioma Genome Atlas
CXCL12	C-X-C motif chemokine ligand 12
DETC	sodium diethyldithiocarbamate trihydrate
DHE	dihydroethidium
DMEM	Dulbecco’s modified Eagle’s medium
EGFR	epidermal growth factor receptor
EGFRvIII	epidermal growth factor receptor variant III
EV	empty vector
GBM	glioblastoma
H_2_O_2_	hydrogen peroxide
HgCl_2_	mercuric chloride
HNE	4-hydroxy-2-nonenal
mRNA	messenger RNA
OXPHOS	oxidative phosphorylation
PBS	phosphate-buffered saline
p-AKT	phosphorylated AKT
p-EGFR	phosphorylated EGFR
qRT-PCR	quantitative real-time polymerase chain reaction
ROS	reactive oxygen species
SDH	succinate dehydrogenase
SDHA	succinate dehydrogenase subunit A
SDHAF	succinate dehydrogenase assembly factor
SDHB	succinate dehydrogenase subunit B
SDHC	succinate dehydrogenase subunit C
SDHD	succinate dehydrogenase subunit D
shRNA	short hairpin RNA
SOD2	superoxide dismutase 2
TCGA	The Cancer Genome Atlas
TMZ	temozolomide
WHO	World Health Organization
