# Supplementary material for: Succinate Dehydrogenase Subunit D as a Redox-Responsive Mitochondrial Component Linked to Aquaporin-Associated Hydrogen Peroxide Signaling in Glioblastoma Cells
Source: Antioxidants (Basel). 2026 Sep 5;15(9):1125. doi: 10.3390/antiox15091125 (PMC13603557; doi:10.3390/antiox15091125)
Supplement: Supplementary file 1 [file antioxidants-15-01125-s001.zip › antioxidants-4437543-supplementary.pdf]

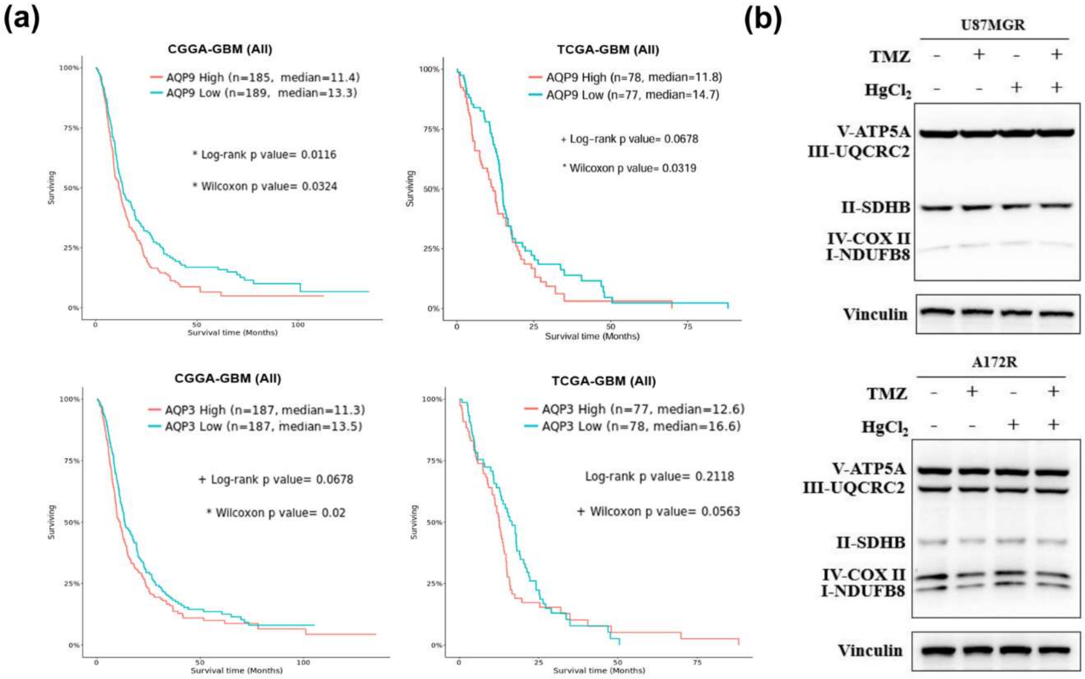
(a)
CGGA-GBM (All)
AQP9 High (n=185, median=11.4)
AQP9 Low (n=189, median=13.3)
* Log-rank p value= 0.0116
* Wilcoxon p value= 0.0324
TCGA-GBM (All)
AQP9 High (n=78, median=11.8)
AQP9 Low (n=77, median=14.7)
+ Log-rank p value= 0.0678
* Wilcoxon p value= 0.0319
CGGA-GBM (All)
AQP3 High (n=187, median=11.3)
AQP3 Low (n=187, median=13.5)
+ Log-rank p value= 0.0678
* Wilcoxon p value= 0.02
TCGA-GBM (All)
AQP3 High (n=77, median=12.6)
AQP3 Low (n=78, median=16.6)
Log-rank p value= 0.2118
+ Wilcoxon p value= 0.0563
Surviving
Survival time (Months)
(b)
U87MGR
TMZ
HgCl2
V-ATP5A
III-UQCRC2
II-SDHB
IV-COX II
I-NDUFB8
Vinculin
A172R

Figure S1

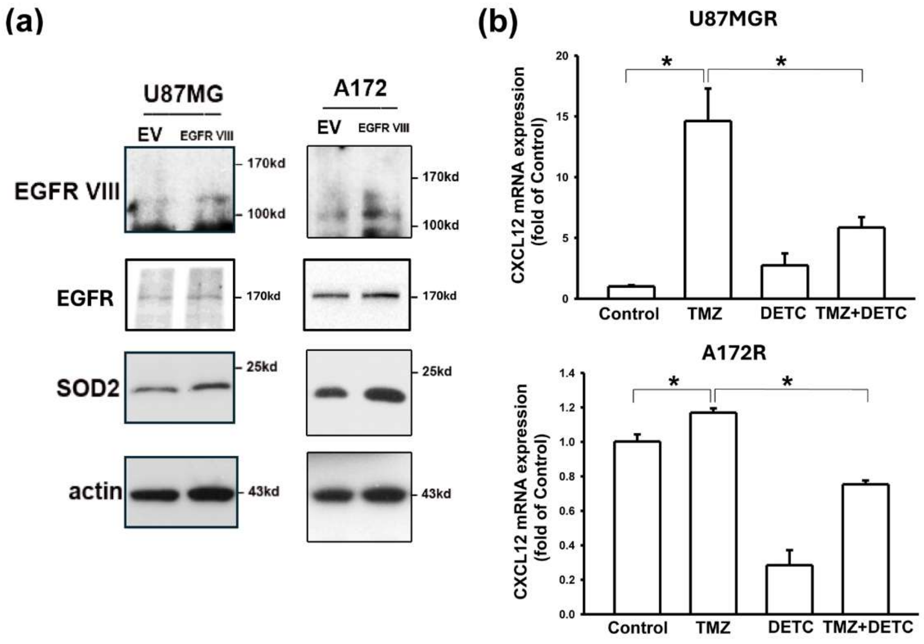
(a)
U87MG
EV
EGFR VIII
A172
EV
EGFR VIII
EGFR VIII
170kd
100kd
EGFR
170kd
SOD2
25kd
actin
43kd
(b)
U87MGR
CXCL12 mRNA expression (fold of Control)
Control
TMZ
DETC
TMZ+DETC
A172R
CXCL12 mRNA expression (fold of Control)
Control
TMZ
DETC
TMZ+DETC

Figure S2
